# Colostrum avoidance and associated factors among mothers of less than 6-month-old children in Dilla town, Southern Ethiopia

**DOI:** 10.3389/fped.2024.1399004

**Published:** 2024-07-11

**Authors:** Anteneh Gashaw, Daniel Kebede, Teferi Regasa, Hermela Bekele

**Affiliations:** Department of Midwifery, College of Medicine & Health Sciences, Dilla University, Dilla, Ethiopia

**Keywords:** colostrum, early initiation of breastfeeding, breastfeeding practices, infant nutrition, prelacteal feeding, Ethiopia

## Abstract

**Background:**

Colostrum, often referred to as “golden milk,” is the initial milk produced after birth, crucial for preventing childhood malnutrition and boosting natural immunity. Neglecting colostrum intake heightens vulnerability to infections and mortality, particularly in developing regions of the world. Colostrum avoidance is prevalent in Ethiopia. This research aims to investigate the frequency and factors that contribute to colostrum avoidance in Dilla town, Southern Ethiopia.

**Method:**

A community-based, cross-sectional study was carried out in late 2023. Data were gathered from 350 participants, selected through multistage sampling, using structured questionnaires and face-to-face interviews. Data entry was conducted using Epi Data version 4.2.0 and transferred to Stata version 18 for analysis. Bivariate and multivariable logistic regression analyses were employed to identify the factors associated with colostrum avoidance, with a significance level of *p* < 0.05.

**Result:**

The rate of prevalence of colostrum avoidance was 28.3% [95% confidence interval (CI): 18.4%–38.2%]. Late initiation of breastfeeding [adjusted odds ratios (AOR) 4.15, 95% CI: 2.51–6.84] prelacteal feeding, non-use of postnatal care (PNC) (AOR 1.79, 95% CI: 1.05–3.04), and lack of information about colostrum (AOR 1.88, 95% CI: 1.14–3.08) were the factors significantly associated with colostrum avoidance.

**Conclusions and recommendations:**

A high prevalence of colostrum avoidance was observed, with prelacteal feeding, delayed initiation of breastfeeding, lack of PNC, and inadequate maternal knowledge about colostrum identified as contributing factors. Consequently, interventions aimed at reducing colostrum avoidance should prioritize improving access to and promotion of PNC, promoting timely initiation of breastfeeding, and intensifying awareness campaigns highlighting the advantages of colostrum, while emphasizing the risks associated with prelacteal feeding.

## Background

One of the 15 selected preventive survival measures for children involves promoting optimal feeding practices for infants and young children (1, 2). Breastfeeding (BF), or *yetut wetet*, is considered a proven method for ensuring the survival of toddlers, and the World Health Organization (WHO) recommends the initiation of breastfeeding within the first hour after birth (3).

The initial years of a toddler's life are crucial for their growth and overall development (1, 2). Breastfeeding is considered a fundamental entitlement for children and plays a critical role in their survival, preventing childhood illnesses and providing optimal nutrition for the newborn (4, 5). It has significant positive impacts on the immune system, cognitive development, economic factors, and environmental aspects. In addition, breastfeeding substantially reduces the risk of obesity, type 2 diabetes, and other related chronic non-communicable diseases in children (2). Moreover, breastfeeding has been demonstrated to aid women in weight loss and reduce their likelihood of experiencing hemorrhaging, postpartum depression, breast cancer, ovarian cancer, and endometrial cancer (5).

The initial liquid, known as colostrum (Inger), which is thick, sticky, and clear to yellowish in color, transports proteins, vitamin A, and maternal antibodies until lactation is well-established (6). It is secreted around the end of pregnancy and in the early postpartum period (7). Lactose levels in colostrum are lower than those in mature milk, but they tend to rise over time (8). Colostrum is considered a baby's first vaccine, and feeding an infant helps foster intelligence (9). In spite of these benefits, in some settings, colostrum is seen as being heavy, thick, filthy, toxic, and harmful to children's health; as a result, in some communities, a portion of colostrum is rejected (9). Colostrum avoidance is considered to occur at some point during the first 5 days following childbirth (2). Colostrum avoidance includes not initiating breastfeeding within the first hour of birth, pumping and throwing away colostrum, and/or wet nursing (10).

Colostrum avoidance is a widespread global practice, with significant rates observed in various settings. For instance, in Uttarakhand, India, 92% of mothers dispose of colostrum, while in Nepal, the figure is 16.5%, in Pakistan, it is27.9%, and in Burkina Faso, it is 16% (11, 12). Similarly, in Ethiopia, the practice of discarding colostrum is prevalent. In towns and regions such as Afar, Debre Tabor, Raya Kobo, Axum, and Motta, the percentages of mothers reporting colostrum disposal are 35%, 25.6%, 13.5%, 6.3%, and 20.3%, respectively (4, 6, 13, 14).

A global study published in *The Lancet* in 2023 revealed that only 48% of infants and young children worldwide are breastfed in accordance with WHO (World Health Organization) recommendations (15). Due to the lack of colostrum within the first hour of birth, more than 4,000 toddlers and young children die annually (16). In Ethiopia, neonatal mortality remains a significant health concern. Despite the known benefits of colostrum feeding and exclusive breastfeeding (EBF) in reducing newborn morbidity and mortality (16, 17), approximately 27% of infants in Ethiopia do not receive colostrum within 1 h of birth (16, 17). Mothers who breastfeed lack an understanding of colostrum feeding, both in high-income and low- and middle-income nations (16).

Despite efforts being made by the authorities concerned, neonatal mortality rates in Ethiopia remain high, with little change noted between 2016 (29/1,000 live births) and 2019 (30/1,000 live births) (18). Factors contributing to colostrum avoidance include home deliveries, lack of antenatal care (ANC) attendance, and limited knowledge of breastfeeding best practices (2). In addition, 27% of infants receive prelacteal feeds within the first 3 days, especially in rural areas. Studies show colostrum avoidance rates varying from 6% to 76.9% (2, 18). Although breastfeeding is prevalent, the rate of exclusive breastfeeding at 6 months stands at only 59% (18).

Against this background, the aim of the study is to investigate the practice of colostrum avoidance and its associated factors among mothers with children under 6 months of age in Dilla town, Gedeo Zone, Southern Ethiopia. This research seeks to understand the prevalence of colostrum avoidance in the area and identify the factors that contribute to this practice among mothers. By examining these factors, the study aims to provide insights that can inform interventions and strategies directed at promoting optimal breastfeeding practices and improving infant health outcomes in the region.

## Methods

### Study area and period

A community-based cross-sectional study was undertaken in Dilla town, the capital of Gedeo Zone within the Southern Nations, Nationalities, and People’s Region (SNNPR). Dilla town covers an area of 135 km^2^ and is characterized by a *woyinedega* climate (a high-altitude zone characterized by moderate temperatures and rainfall). The town comprises five *kebeles* (the smallest administrative unit in the country's government structure): Sesa, Odaya, Haroresa, Haro Welabu, and Chichu. Dilla has a total population of 96,920. Its facilities include a general hospital, two health centers, private clinics, pharmacies, a university, private colleges, preparatory and high schools, elementary and junior schools, a post office, and telecommunication services. This study was conducted between 5 September and 6 October 2023.

### Population

The source of population for this study was all women who had given birth in the last 6 months in Dilla, and the study population for this study was all mothers who had been residing in the selected *kebeles* and had a child less than 6 months of age prior to the study period.

### Eligibility criteria

The inclusion criteria for this study encompassed mothers with infants under 6 months old and who had been residing in the area for at least 6 months. The exclusion criteria involved mothers who were critically ill, unable to participate in the study, or unavailable during three consecutive visits to the selected *kebeles* during the data collection period.

### Sample size determination

Sample size was determined using a single population proportion formula by considering the following assumption:

$$n={\left(z_{\alpha }/2\right)}^{2}p(1-p)/w^{2}$$

✓ The proportion of colostrum avoidance in the Afambo district of the Afar Regional State was 35% (*p* = 0.35).
✓ 95% level of confidence (z = 1.96).
✓ 5% of marginal error (w = 0.05).

Here, *n* = (z*_α_*_/2_)^2^p (1 − p)/w^2^.

$$n=(1.96{)}^{2}*0.35(1\text{–}0.35)/(0.05{)}^{2}$$

n=(3.8416)0.35(0.65)/(0.0025)

n=0.873964/0.0025

n=349.5≈350

After adding a 10% non-response rate, the total sample size was 385.

### Sampling technique

The study employed a two-stage sampling technique. In Dilla town, which comprises a total of five *kebeles*, a subset of four *kebeles* (Bareda, Haroke, Tena Tabiya, and Bedecha) was randomly selected using a simple random sampling method. Subsequently, all households within these selected *kebeles* containing mothers with children aged less than 6 months were numbered. The total sample size was then proportionally allocated to each selected *kebele*, and a systematic sampling technique was applied to choose study participants. During data collection, one eligible mother with a child aged less than 6 months was selected from each household unit. In cases where more than one potential respondent resided in a household, a simple random sampling method was employed to make the final selection.

### Variables

The dependent variable in this study was colostrum avoidance, while the independent variables encompassed various sociodemographic characteristics, including age, marital status, occupational status, household composition, and ethnicity. In addition, factors related to previous breastfeeding practices such as delayed initiation of breastfeeding, exposure to formula feeding advertisements, and prelacteal breastfeeding were examined. Maternal obstetrics and health-related conditions such as ANC visits, breastfeeding counseling during ANC visits, place and mode of delivery, postnatal care (PNC) visits, birth attendant, and presence of medical illnesses were also considered. Furthermore, the level of knowledge about colostrum was assessed as an additional variable.

### Operational definitions

Colostrum avoidance: the failure to feed infants the ﬁrst, thick, and yellowish milk that is produced in the ﬁrst 3 days after birth (10).

Prelacteal feeding: if an infant within the first 3 days of life is fed something other than breast milk (19).

Delayed initiation of BF/delayed lactation: initiation of BF more than 1 h after birth (19).

Insufficient milk secretion: a condition in which a lactating mother produces less breast milk than is needed to meet the nutritional demands of her infant whether immediately or late postpartum.

Attitude toward colostrum feeding: this is assessed through a 5-point Likert scale (i.e., a score of 1 for strongly disagree, 2 for disagree, 3 for neutral, 4 for agree, and 5 for strongly agree); it is then dichotomized by a mean score as favorable vs. viewed unfavorably (10).

Knowledge: if respondents correctly answer >60% of knowledge questions related to colostrum feeding, they are considered to have good knowledge, and those who fall below that percentage have poor knowledge (10).

### Data collection procedure

Data were collected by holding face-to-face interviews and using structured questionnaires, which were filled by data collectors. The questionnaires were prepared by considering the local situation of the study area and the purpose of the study. Initially, they were written in English and then translated into Amharic. The interviews were conducted by data collectors.

### Data quality control

To maintain quality of the data, the questionnaires were pretested in 19 individuals (5% of the samples) selected from a nearby *kebele* to gauge the accuracy of the responses and to estimate the time needed for holding the interviews. Based on the pretest, appropriate modifications were made before the actual data collection exercise. Each day, the collected data were checked for completeness and consistency. The investigator communicated with the supervisors on a daily basis. Interviews were conducted in a private and confidential manner. The collected data were reviewed and checked for completeness before data entry was undertaken.

### Data processing and analysis

After the data collection process was completed, the data were cleaned, coded, and entered into Epi Data version 4.2.0. Subsequently, they were exported to Stata version 18 for further analysis. Descriptive statistics such as tables, graphs, and charts were used to describe the characteristics of the study participants. Bivariable binary logistic regression was fitted to identify the factors associated with colostrum avoidance. Variables with a *p*-value of ≤0.2 in the bivariable analysis were fitted to the multivariable logistic regression analysis. Both crude (COR) and adjusted odds ratios (AOR) with the corresponding 95% confidence intervals (CIs) were calculated to show the strength of association. In the multivariable analysis, variables with a *p*-value of <0.05 were considered statistically significant.

## Result

### Sociodemographic characteristics

The responses of 350 study participants were recorded, yielding a response rate of 90.9%. The mean age of the study participants was 27.3 (±5.63) years. Among the total respondents, 258 (73.8%) were married. The majority of the study participants were followers of the Protestant religion and Gedeo in ethnicity, numbering 246 (70.3%) and 293 (83.6%), respectively. Out of the 350 participants, 33.7% were not employed outside the home. Approximately 126 respondents (36%) obtained broadcasting information via radio about the benefits of colostrum feeding and the consequences of not providing colostrum to newborns (Table 1).

**Table 1:** Sociodemographic characteristics of mothers of children less than 6 months old in Dilla town, Gedeo Zone, Southern Ethiopia, 2023 (*n* = 350).

Variable	Frequency (*n* = 350)	Percentage
Age		
18–26	102	29.2
27–38	237	67.8
39–49	11	3
Marital status		
Married	258	73.8
Single	26	7.4
Divorced	29	8.3
Widowed	37	10.5
Religion		
Orthodox	43	12.2
Protestant	246	70.3
Catholic	17	4.9
Muslim	36	10.3
Other	8	2.3
Ethnicity		
Gedeo	293	83.6
Sidama	24	6.9
Oromo	23	6.6
Other	10	2.9
Educational status of the mother		
Unable to read and write	69	19.8
Can read and write without formal education	24	6.9
Elementary	114	32.6
High school	88	25.2
College/University and above	54	15.5
Mother’s occupation		
Not employed outside the home	118	33.7
Employed outside the home	232	**66** **.** **3**
Educational status of the father		
Unable to read and write	23	6.6
Can read and write without formal education	60	17.1
Elementary	71	20.3
High school	104	29.7
College/University and above	92	26.3
Father’s occupation		
Civil servant	76	21.7
Trader	166	47.4
Daily laborer	80	22.9
Other	28	8

### Maternal healthcare service utilization–related factors

In this study, the majority of mothers (279, 79.7%) received ANC, 185 (52.8%) utilized ANC at least four times, or equal to four times, and 248 (70.9%) underwent BF counseling during ANC. A total of 161 (46%) mothers had birth spacing greater than 24 months. The majority of mothers gave birth through vaginal delivery (326 or 93.1%) and 230 at a health center (65.7%). Among the 350 study participants, 299 had PNC (85.4%) and 62 (17.8%) had not experienced any neonatal illness (Table 2).

**Table 2:** Healthcare service utilization and obstetric history of mothers having children less than 6 months of age in Dilla town, Gedeo zone, Southern Ethiopia, 2023 (*n* = 350).

Variable	Frequency (*n* = 350)	Percentage
ANC follow-up		
No	71	20.3
Yes	279	79.7
Frequency of ANC follow-up		
1	27	7.7
2	36	10.3
3	102	29.2
≥4	185	52.8
BF counseling during ANC		
No	102	29.1
Yes	248	70.9
Birth spacing (months)		
<24	189	54
>24	161	46
Mode of delivery		
Vaginal	326	93.1
Cesarean section	24	6.8
Place of delivery		
At home	70	20
At health facilities	280	80
PNC follow-up		
No	51	14.6
Yes	299	85.4

### Colostrum avoidance

The prevalence rate of colostrum avoidance within 3 days after delivery among mothers having children less than 6 months of age was 28.3% (95% CI: 18.4–38.2). The primary reason given by the respondents for colostrum avoidance was that colostrum constitutes a dirty portion of breast milk (43 or 12.4%). It was found that 120 (34.2%) mothers gave prelacteal feeding for their neonates. Among mothers who gave prelacteal feeding, 30 (8.4%) gave water. The main reason given by them for their practice was that they had inadequate breast milk (76 or 21.7%. Out of the 350 respondents, 298 (85.1%) started BF within 1 h after delivery (Table 3).

**Table 3:** Breastfeeding practice–related characteristics of study participants of mothers having children less than 6 months of age in Dilla town, Gedeo Zone, Southern Ethiopia, 2023 (*n* = 350).

Variable	Frequency	Percentage
Practice of colostrum avoidance (*n* = 350)		
No	251	71.7
Yes	99	28.3
Reason to avoid (*n* = 99)		
Cause illness to neonates	13	13.13
Culturally forbidden	15	15.15
Dirty portion of breast milk	43	43.43
Not good for neonate health	6	6.06
Very thick	14	14.14
Not good for neonate growth and development	8	8.08
Practice of prelacteal feeding (*n* = 350)		
No	230	65.8
Yes	120	34.2
Type of prelacteal feeding (*n* = 120)		
Water	30	25
Butter	45	37.5
Honey	7	5.83
Cow milk	35	29.16
Tea	3	2.5
Reason for prelacteal feeding (*n* = 120)		
Inadequate breast milk secretion	59	49.16
Delayed lactation	34	28.33
Cultural practice	17	14.16
Maternal illness	7	5.83
Neonatal feeding problem	3	2.5
Time of BF initiation (*n* = 350)		
Within 1 h	298	85.1
After 1 h	52	14.9

### Knowledge and information about colostrum

Out of the total study participants, 256 (73.3%) had heard about colostrum, and the main source of their information was health professionals (196 or 56%). Among the 350 respondents, those who said that the color of colostrum is yellow were 303 (86.6%), and the number of respondents who knew that it can protect the neonate from infectious diseases was 213 (60.9%). The number of mothers who said that it was an important part of breast milk and that it is good practice to feed colostrum to the baby even when the mother is sick was 215 (61.4%) and 210 (60%), respectively. The majority of mothers(199 or 56.9%) responded that it was good to feed colostrum even when the baby is sick (Table 4).

**Table 4:** Knowledge and information–related characteristics of study participants of mothers having children less than 6 months of age in Dilla town, Gedeo Zone, Southern Ethiopia, 2023 (*n* = 350).

Variable	Frequency (*n* = 350)	Percentage
Have you heard about colostrum?		
No	94	26.7
Yes	256	73.3
Source of information		
Health professionals	196	56
Family	107	30.5
Media	15	4.3
Friends	32	9.2
Color of colostrum		
Yellow	303	86.6
Red	21	5.9
Other*	26	7.5
Can protect the neonate from infectious disease		
No	137	39.1
Yes	213	60.9
It is an important part of breast milk		
No	135	38.6
Yes	215	61.4
Feeding is good even when the mother is sick		
No	140	40
Yes	210	60
Feeding is good even when the baby is sick		
No	151	43.1
Yes	199	56.9
Attitude		
Favorable	150	42.85
Unfavorable	200	57.14

*Mothers who stated that the color of colostrum is blue, black or brown.

### Factors associated with colostrum avoidance

In the bivariable analysis, occupational status of the husband, place of birth, breastfeeding initiation time, prelacteal feeding, PNC utilization, and information about colostrum were candidate variables for a multivariable logistic regression analysis. Whereas in the final model timing of initiation of breastfeeding, prelacteal feeding, PNC service, and having information about colostrum were associated with colostrum feeding, those mothers who initiated breastfeeding more than one hour after delivery were nearly four times more likely to discard colostrum compared with those who initiated breastfeeding within 1 h after delivery (AOR 4.15, 95% CI: 2.51–6.84). Mothers who gave prelacteal feeds were nearly three times more likely to discard colostrum compared with those who did not practice prelacteal feeding (AOR 3.16, 95% CI: 1.93–5.15). The odds of discarding colostrum among mothers who did not attend PNC services were 1.79 times (95% CI: 1.05–3.04) higher than those who used PNC services. The likelihood of discarding colostrum was higher among mothers who had no information about colostrum (AOR 14.795, 95% CI 5.567–39.314) compared with their more informed counterparts (Table 5).

**Table 5:** Factors associated with colostrum avoidance of mothers having children less than 6 months of age in Dilla town, Gedeo Zone, Southern Ethiopia, 2023 (*n* = 350).

Variables	Category	Colostrum avoidance		COR (95% CI)	AOR (95% CI)
		Yes	No		
Prelacteal feeding	Yes	31	64	4.89 (3.13–7.65)*	3.16 (1.93–5.15)*
	No	23	232	1	1
PNC utilization	Yes	29	225	1	1
	No	25	71	2.73 (1.78–4.29)*	1.79 (1.05–3.04)*
Time of initiation of BF	Within 1 h	26	253	1	1
	After 1 h	28	43	6.33 (3.87–9.70)*	4.15 (2.51–6.84)*
Information about colostrum	No	54	40	10.56 (4.960–21.749)	14.795 (5.567–39.314)*
	Yes	29	227	1.00	1.00

* *p*< 0.001.

## Discussion

The findings of this study reveal that in Dilla town, 28.3% (95% CI: 18.4%–38.2%) of mothers with infants under 6 months opted to avoid giving colostrum. This result is more or less consistent with that of research conducted in Afambo district, Ethiopia (20), which is that approximately 35% of mothers were discarding colostrum, and also with that of the study conducted in India (1), where 23% of participating mothers were found to practice colostrum avoidance. The aforementioned rate is notably higher than that of the findings from other regions, such as Aksum town in Ethiopia (6.3%) (4), Raya Kobo district (13.5%) (6), Bure district in Ethiopia (14.5%) (21), and Chencha district in Southern Ethiopia (15.3%) (22). The observed disparities could stem from variations in sample size, geographical factors, and the age range of children included in the study, potentially introducing recall bias.

The study found that only 28.3% of mothers avoided colostrum, which is significantly lower than the reported 92% avoidance rate among the Khos tribal community of Uttarakhand, India (12). This difference could be explained by sociocultural differences in breastfeeding practices, variances in access to maternal healthcare services, and the geographical distribution of the study participants. These variations highlight the intricate relationship between cultural norms, socioeconomic status, and geographical factors influencing colostrum feeding practices among mothers with infants under 6 months of age.

According to the findings of the current study, one factor linked with colostrum avoidance is the timing of the initiation of breastfeeding. Mothers who started breastfeeding more than an hour after delivery were 4.15 times more likely to discard colostrum compared with those who began breastfeeding within the first hour after delivery. This aligns with that of a previous study conducted in Raya Kobo, Northern Ethiopia (6), and a study conducted in a rural part of Pakistan (23). The reasoning could be that mothers who discard colostrum might take a longer time to initiate breastfeeding, or it could be that mothers who delay breastfeeding may have less oversight from healthcare professionals, allowing them the opportunity to discard colostrum without intervention. In both the specific area under study and across Ethiopia as a whole, there is a significant requirement for interventions that are focused on exactly when breastfeeding begins. This implies that there may be factors influencing the timing of breastfeeding initiation that need attention, such as cultural practices, healthcare practices, or maternal knowledge and support (22). By addressing these factors through targeted interventions, the potential to improve breastfeeding practices and, as a consequence, enhance maternal and infant health outcomes, arises.

This study also found that mothers who provided prelacteal foods to their babies were more inclined to avoid colostrum compared with those who did not offer such foods. Those who practiced prelacteal feeding were 3.16 times more likely to avoid colostrum compared with those who did not. This mirrors findings from previous studies conducted in the North Wollo Zone (6), Aksum town (4), and Chencha district in Ethiopia (22). The aforementioned behavioral characteristics of mothers may stem from the belief that prelacteal foods are beneficial for the health of their infants. Furthermore, mothers may fear that giving colostrum to their newborns could lead to diarrhea and stomach discomfort. Raising awareness about the disadvantages of prelacteal feeding and the importance of colostrum feeding is essential in the study area. This need for awareness is supported by previous research findings (24).

In this study, the level of information about breastfeeding was identified as a factor associated with colostrum avoidance. Mothers who lacked information about breastfeeding were 14.7 times more likely to avoid colostrum compared with those who were more informed about breastfeeding. This finding aligns with those of studies conducted in Raya Kobo district (6), the Shewa region of Ethiopia (25), and Pakistan (23), where mothers who had less awareness of the benefits of colostrum were more likely to avoid it compared with those who were more informed about its advantages for infants. In Ethiopia, emphasizing key messages about breastfeeding during healthcare visits, rather than solely in the study area, has been shown to positively influence the avoidance of colostrum. This conclusion was supported by research conducted in Chencha district (22).

On the other hand, the utilization of PNC services was significantly associated with less colostrum avoidance. This finding was consistent with that of a study conducted in Axum town (4) and Chencha district in Ethiopia (22). This might be attributed to the fact that mothers who attend PNC services might have access to the counseling sessions conducted by healthcare workers on the importance of colostrum feeding, and therefore, they are more likely to provide colostrum feeding to their newborns. This suggests that in the study area, it is crucial to enhance the utilization of PNC services and improve the quality of counseling provided during these visits.

Many participants have attended ANC sessions and received information about breastfeeding, yet a significant number of them avoid colostrum. This suggests potential issues with the quality of ANC follow-up and the content of counseling. Therefore, future researchers should focus on evaluating the quality of ANC follow-up and counseling specifically related to breastfeeding in the study area.

This study has limitations, including recall bias due to participants’ difficulty in remembering events from the past 6 months and the absence of qualitative data supplementation. Moreover, it shares the inherent limitations of cross-sectional study designs. To address these issues in future research, immediate data collection methods or longitudinal studies could mitigate recall bias, while integrating qualitative data could provide deeper insights into the factors influencing colostrum avoidance and breastfeeding practices. In addition, exploring alternative study designs, such as cohort studies, could offer a more comprehensive understanding of colostrum feeding practices. Future research could also focus on evaluating interventions aimed at promoting colostrum feeding and improving breastfeeding practices, with tailored community-based educational programs or healthcare interventions addressing specific barriers to colostrum feeding and highlighting the benefits of the practice. The association between knowledge and attitudes about colostrum avoidance can also be assessed to formulate appropriate strategies.

## Conclusion

This study revealed a substantial percentage of occurrence of colostrum avoidance, highlighting its significance in the context of breastfeeding practices. Several factors were found to be closely linked to this trend. Among these factors were the timing of initiating breastfeeding, the practice of prelacteal feeding, the utilization of postnatal care services, and the level of awareness about the importance of colostrum feeding. The results indicate that interventions targeting these factors could potentially lead to a reduction in colostrum avoidance and promote better breastfeeding practices in the studied population.

## Data Availability

The raw data supporting the conclusions of this article will be made available by the authors without undue reservation.
